# Celiac disease screening in children: evaluating the evidence, benefits, and challenges

**DOI:** 10.3389/fped.2025.1562073

**Published:** 2025-04-03

**Authors:** Maria Naredi Scherman, Jessica Melin, Daniel Agardh

**Affiliations:** Celiac Disease and Diabetes Unit, Department of Clinical Sciences, Lund University, Malmö, Sweden

**Keywords:** celiac disease, screening, children, transglutaminase antibody, HLA, quality of life, costeffectiveness, population

## Abstract

Comprehensive screening of the general population is the only approach capable of identifying the majority of cases with celiac disease. In 2023, the Italian Parliament enacted a law to implement nationwide screening for celiac disease and type 1 diabetes. However, critical decisions regarding the target population, optimal timing, and screening methods remain unresolved. Previous observational studies on birth cohorts of children with genetic risk for these conditions have demonstrated that the incidence peaks early in life and is influenced by HLA risk genotypes. This mini-review explores different aspects of screening for celiac disease, presenting the advantages and challenges of identifying children before onset of symptoms. In addition, we summarize the current knowledge and gaps in understanding related to screening programs for celiac disease in children and adolescents and discuss health benefits, psychosocial aspects and cost-effectiveness, and their potential implications for future public health strategies.

## 1 Introduction

Advancements in technology have significantly improved the feasibility of mass screening for immune-mediated diseases. Celiac disease is a chronic autoimmune disorder that targets the mucosa of the small intestine in genetically predisposed individuals carrying the human leukocyte antigen (HLA) risk genotypes DQ2 and/or DQ8. Its pathogenesis is strongly associated with elevated levels of tissue transglutaminase (tTG) autoantibodies (1, 2), detectable in the blood long before the clinical onset of the disease (3) and thereby making it a valuable marker for screening (4).

The primary rationale for screening for celiac disease is early detection and treatment with gluten-free diet (GFD), which may prevent long-term complications. According to the World Health Organization (WHO), the ten criteria established by Wilson and Jungner must be considered when evaluating a condition for screening (5). Celiac disease meets several of these criteria: it is an important health problem with a latent or early symptomatic stage, there is an appropriate test available, and an accepted treatment for affected individuals. However, certain criteria remain subject to debate. Particularly, the progression from latent to clinically manifest disease is not yet fully understood, consensus is lacking regarding the criteria for determining which individuals should receive treatment, and the cost-effectiveness of systematic screening relative to overall healthcare costs remains uncertain. Despite ongoing debate regarding whether celiac disease meets the criteria for general population screening (6–9), Italy has taken a pioneering step by approving a law to implement nationwide screening for celiac disease and type 1 diabetes in children aged 1–17 years (10).

Celiac disease has been described as a clinical “chameleon” due to its wide range of presentations, varying from severe malabsorption to nearly asymptomatic cases (11, 12). In children, gastrointestinal symptoms and malabsorption are more common, whereas adults often present with extraintestinal manifestations (13). This complexity often results in diagnostic delays spanning months to years (14–18), yet most individuals remain undiagnosed unless active screening is performed (19). The key questions that arise are whether children should be screened for celiac disease and what the potential benefits and drawbacks of such an approach might be?

## 2 To screen or not to screen?

### 2.1 What are the health benefits of screening?

Celiac disease is among the most prevalent autoimmune conditions in children, with a global prevalence of approximately 1% (20). However, recent screening studies in children and adolescents suggest a higher prevalence of up to 3% in certain populations (21–23). Despite increased awareness and improved detection, a significant proportion of cases remain undiagnosed without mass screening; a recent study found that 60% of cases went undetected in the absence of systematic screening (23).

The clinical manifestations of celiac disease exhibit a remarkable variation. While some patients present with classic symptoms of malabsorption, including diarrhea, steatorrhea, weight loss, and growth retardation, others develop more non-specific gastrointestinal symptoms, such as abdominal pain and constipation (24), or extraintestinal symptoms or signs such as iron deficiency, short stature, delayed puberty, osteoporosis, infertility, dermatitis herpetiformis, enamel defects, neurological issues (e.g., gluten ataxia, peripheral neuropathy), or psychiatric symptoms (12, 25). Celiac disease is also associated with several other conditions such as selective immunoglobulin A (IgA) deficiency, type 1 diabetes, autoimmune thyroid disease and psoriasis, as well as chromosomal abnormalities such as Down, Turner, and William syndromes (25, 26). Given these known associations, celiac disease should always be considered in patients with these conditions.

The wide spectrum makes celiac disease challenging to diagnose, often leading to significant delay in diagnosis. The delay can range from months to over a decade (14–18) and patients are frequently misdiagnosed with alternative diagnoses such as anemia, irritable bowel syndrome, or stress-related disorders, before celiac disease is identified (14). In children, the diagnostic delay tends to be shorter than in adults. One study reported a median delay of five months (17), while another documented a broader range of 2–109 months (27). Prolonged delay in diagnosis is associated with more severe disease at presentation, including higher tTG autoantibody levels, more advanced villous atrophy, persistent symptoms, and reduced quality of life (18, 28). Interestingly, approximately one-fifth of children with screening-detected celiac disease had sought medical care for symptoms prior to screening, but the possibility of celiac disease had not been considered (29).

A controversial issue in screening is whether children identified as asymptomatic benefit from a diagnosis. In an Italian school-based screening program, as many as two-thirds of the children diagnosed with celiac disease were reportedly asymptomatic (30). However, studies have shown that many “asymptomatic” individuals have subtle symptoms or laboratory abnormalities that improve on a GFD (8). Furthermore, screening-detected and clinically detected children do not differ in regards of autoantibody titers, histological damage, or response to GFD (31). It has also been shown that screening-detected children exhibit systemic inflammation at diagnosis which resolves with dietary intervention, indicating that also these children benefit from treatment (32).

Previously undetected anemia, low ferritin, and other micronutrient deficiencies that improve after starting a GFD have been observed in screening-detected children (33, 34). Poor growth and reduced body mass index (BMI) are also common (33, 35). Dietary intervention leads to significant improvement (33), emphasizing the importance of early detection to prevent long-term irreversible effects on stature.

Another critical consideration is bone health. Malabsorption of calcium and vitamin D can result in osteopenia, osteoporosis, and increased fracture risk (12, 36, 37). Childhood and adolescence are pivotal periods for bone accretion and the peak bone mass reached in young adulthood is predictive for the risk of osteoporosis later in life (38). Early diagnosis and adherence to a GFD during these critical periods have been shown to normalize bone mineral density and improve the overall outcomes of bone health (27, 32, 39), which is not seen to the same extent in individuals diagnosed as adults (40).

In a long-term perspective, early diagnosis and adherence to a GFD during critical developmental periods not only optimize growth and bone health outcomes but also mitigate the risk of severe complications, including certain malignancies. Celiac disease has been associated with an increased risk of enteropathy-associated T-cell lymphoma (EATL) and intestinal adenocarcinomas, and although these malignancies are rare, it underlines the importance of early diagnosis and adherence to a GFD to reduce cancer risk (41, 42).

These findings highlight the potential benefits of screening programs for celiac disease. Early diagnosis can reduce nutrient deficiencies, improve growth, enhance bone health, and potentially prevent severe complications such as malignancies. But do the benefits of widespread screening outweigh the associated challenges and costs?

### 2.2 Which individuals should be targeted for screening?

Symptomatic screening for celiac disease involves individuals seeking medical care for classical symptoms such as chronic diarrhea, abdominal pain, or failure to thrive. These symptoms are common in the general pediatric population, and most children presenting with such complaints do not have celiac disease. Studies have shown that symptom-based screening is a poor discriminator of celiac disease, as there is no significant difference in symptom prevalence between children who test positive or negative through screening (23, 43, 44).

Expanding screening to include individuals with extraintestinal manifestations, abnormal laboratory findings, or those belonging to risk groups significantly increases diagnostic yield. Individuals at increased risk of developing celiac disease are first-degree relatives, patients with other autoimmune diseases, such as type 1 diabetes or autoimmune thyroid disease, patients with IgA deficiency or psoriasis, or patients with genetic syndromes, such as Down, Turner, or William syndromes. An active case-finding approach is endorsed by both the European Society for Pediatric Gastroenterology, Hepatology, and Nutrition (ESPGHAN) (1, 26) and the North American Society for Pediatric Gastroenterology, Hepatology, and Nutrition (NASPGHAN) (45), which recommend a low threshold for testing individuals within these identified risk groups. However, despite these guidelines, it is estimated that active case-finding would only identify around 40% of cases of celiac disease (43). Although the pooled prevalence of celiac disease among first-degree relatives to affected individuals has been shown to be as high as 7.5% (46), approximately 80%–90% of children with celiac disease autoimmunity or celiac disease detected through mass screening did not have a first-degree relative with the condition (44, 47).

Increased awareness among healthcare providers and the public about the diverse manifestations and related conditions of celiac disease could enhance case detection through active case-finding, but this strategy would still miss most of affected individuals. Comprehensive screening of the general population is the only approach capable of identifying the majority of cases, and in recent years several mass screening programs for celiac disease have been initiated around the world (23, 44, 47). Nevertheless, Italy remains the only country where nationwide screening for celiac disease and type 1 diabetes has been legislatively approved (10).

### 2.3 What are the optimal methods for screening?

In population-wide screening programs, screening tests must exhibit high sensitivity and specificity, as most individuals screened will have a low pre-test probability of disease. Celiac disease screening commonly includes analyzing disease-specific autoantibodies, with IgA endomysial antibodies (EMA) and IgA-tTG showing the highest sensitivity and specificity (11). Due to higher cost, time consuming procedures, and interobserver variability for EMA, IgA-tTG has become the preferred first-line screening method for celiac disease (1, 2, 45). Other autoantibodies, such as antigliadin antibodies (AGA) and deamidated gliadin peptide (DGP) antibodies, are also markers for celiac disease but their lower sensitivity and specificity compared to EMA and IgA-tTG make them unsuitable as first-line screening tests (1, 2, 45). In addition to IgA-tTG, it is generally recommended to take the cost for assessing total IgA in serum in order not to miss celiac disease in individuals with selective IgA deficiency.

An alternative to mass screening with autoantibodies as first-line analysis, is to identify children with increased genetic risk and target screening to these individuals. Celiac disease is strongly associated with HLA genotypes DQ2 and DQ8, and cases in individuals lacking these heterodimers are extremely rare (48). Pre-screening for HLA genotypes effectively excludes individuals without genetic risk from further testing, reducing the number of individuals requiring autoantibody testing. This approach has been implemented in several screening studies, including the multinational TEDDY study (49), the American DAISY study (50), the Swedish CiPiS study (51), and a recent large-scale Italian school-based screening (23). In the Swedish CiPiS study, 3.5% of children carrying HLA risk genotypes DQ2 and/or DQ8 had celiac disease, compared to no cases in the control group lacking any of these HLA risk genotypes (51). Given the shared HLA risk genotypes for celiac disease and type 1 diabetes (52), a joint screening initiative for both conditions could be a practical and cost-efficient alternative. With new technologies for autoantibody detection it is now possible to analyze multiple autoantibodies in a very small volume of blood (53, 54) and combining pre-screening for HLA risk genotypes and subsequent autoantibody testing may improve the efficiency of screening efforts.

### 2.4 When is the optimal time-point to screen?

The timing of screening for celiac disease is important to maximize detection, prevent complications, and avoid unnecessary interventions. Evidence suggests that a significant proportion of cases develop autoantibodies before three years of age (52) making this an important starting point for screening. However, studies have demonstrated that seroconversion can occur well beyond the early years, with new cases emerging up to adolescence (55, 56). Repeated testing every 3–6 years during childhood and adolescence strikes a balance between detecting late-onset cases and minimizing the burden on healthcare resources and families. For children in risk groups, more frequent testing (e.g., every 2–3 years) may be warranted, especially if symptoms or laboratory abnormalities are present. Early adolescence represents a second key time-point period for screening since a delayed diagnosis during late adolescence can lead to more severe complications, including delayed puberty, reduced bone mineral density, and psychological distress. Conversely, children with no identified risk factors and consistently negative IgA-tTG results might require less frequent or no follow-up.

### 2.5 Psychosocial aspects of screening

Even though early detection and treatment of celiac disease can lead to numerous health benefits, adopting a lifelong GFD can pose significant psychosocial challenges, especially for children and adolescents who perceive themselves as asymptomatic. The restrictive nature of a GFD can affect social interactions, particularly in settings where food plays a central role, such as school events, social gatherings, and traveling. These restrictions may lead to feelings of exclusion, embarrassment, or stigma potentially impacting social functioning and overall quality of life (57). For symptomatic individuals, both clinically and screening-detected, quality of life tends to be lower prior to diagnosis but improves after the initiation of a GFD (15, 29). However, in asymptomatic individuals quality of life is comparable to that of healthy peers, both before and after diagnosis (29), indicating that a screening-detected diagnosis does not have a negative impact on quality of life in this group. Furthermore, long-term follow-up of individuals diagnosed in childhood suggest no significant differences in quality of life in adulthood between screening-detected and clinically detected patients (58, 59). Furthermore, despite the potential challenges associated with dietary management, approximately 90% of children and caregivers express satisfaction with participating in screening programs and the subsequent diagnosis of celiac disease (60, 61).

### 2.6 Is screening a cost-effective approach?

The economic burden of celiac disease extends far beyond the costs of maintaining a GFD; it includes healthcare expenses related to diagnostic investigations, follow-up visits, management of complications and associated conditions, and societal costs such as reduced productivity due to work limitations (62). The costs associated with celiac disease vary across different countries, which complicates the estimations of generalizable cost-effectiveness analyses.

A commonly used measure to evaluate the cost-effectiveness of celiac disease screening programs is to estimate the cost per quality-adjusted life year (QALY) gained. A threshold of $50,000 per QALY gained has been widely accepted as the benchmark for cost-effectiveness (63). However, it has been proposed that this threshold may vary based on a country's health expenditure per capita and the life expectancy of the population, with thresholds ranging from less than $100 in low income countries to nearly $100,000 in high income countries (64).

Both the cost-effectiveness of active case-finding in individuals with higher risk of celiac disease, and mass screening in the general pediatric population has been evaluated. A British study found that the cost per QALY for screening newly diagnosed patients with type 1 diabetes ranged from £12,000 to £20,000 (65). A recent Dutch study estimated the QALY gain for active case-finding in a hypothetical cohort of 3-year-old's using point-of-care (POC) tests in children with at least one symptom suggestive of celiac disease (66). This strategy was associated with an additional 4.33 QALYs at a cost of €15,585 compared to current care. This study also assessed the cost-effectiveness of mass screening in the same hypothetical cohort of 3-year-old's, which resulted in an additional 7.46 QALYs at a cost of €28,635 compared to current care (66). A Swedish study based on data from a program involving 12-year-old schoolchildren found a cost of €40,105 per gained QALY (67). These studies indicate that screening for celiac disease is cost-effective, as costs were below the commonly accepted threshold of $50,000 per QALY gained.

However, for screening to be deemed cost-effective, two essential prerequisites must be fulfilled. First, early detection and treatment with GFD must significantly reduce the risk of associated conditions and complications such as osteoporosis and malignancies. Second, screening-detected individuals, including asymptomatic cases, must adhere to the GFD for any health benefits to occur. Concerns have been raised about whether asymptomatic, screening-detected individuals would be sufficiently motivated to comply with the restrictive diet. Some reports have indicated poor adherence rates following mass screening (68, 69), but many studies show that adherence rates in screening-detected children and adults range from 70% to 100% (8, 59), comparable to rates observed in clinically detected patients (31). Importantly, early diagnosis during childhood appears to facilitate long-term adherence to the GFD (29).

It is crucial to recognize that analyses of cost-effectiveness frequently depend on assumptions regarding mortality, morbidity, and adherence to a GFD, and that even minor changes in these assumptions can substantially impact the results of the cost-effectiveness evaluation (6).

## 3 Discussion

Celiac disease meets several of the WHO criteria for public screening (5). Firstly, it affects both children and adults worldwide, with a substantial burden of undiagnosed cases. Secondly, individuals with latent or early symptomatic celiac disease can be identified through reliable autoantibody testing. Thirdly, tests for celiac disease-specific autoantibodies, particularly IgA-tTG, are sensitive, specific, and widely accepted by the population. Finally, treatment with GFD improves symptoms and prevents complications, enhancing quality of life in symptomatic individuals and without deteriorating quality of life in asymptomatic individuals. Despite these factors, several challenges remain before screening for celiac disease can be recommended in the public.

A key question is if screening for celiac disease is cost-effective. Based on available evidence, celiac disease screening programs can be cost-effective, particularly in high-prevalence populations if complications are effectively mitigated and quality of life improved through early detection and treatment. Ensuring adherence to the GFD among screening-detected individuals is essential to maximize the benefit of screening. Further research into optimizing screening strategies and enhancing adherence support will be critical for improving cost-effectiveness and health outcomes.

Moreover, the long-term benefits of diagnosing asymptomatic individuals remain uncertain. As a result, the U.S. Preventive Services Task Force (USPSTF) has determined that there is insufficient evidence to assess the balance of benefits and harms of screening for celiac disease in asymptomatic individuals. This conclusion applies to both mass screening and screening of high-risk groups (7, 70).

Furthermore, the potential psychological impact of false-positive results must be carefully considered, and additional complexity lies in managing individuals with potential celiac disease, i.e., those who test positive for autoantibodies but exhibit normal intestinal mucosa (11). Approximately one-third of such individuals progress to clinical celiac disease, while another third experience normalization of IgA-tTG levels over time (71). There is a potential risk that a positive test result without a diagnosis can increase individuals' anxiety. However, providing information about risk for disease may also raise awareness of symptoms, potentially leading to earlier diagnosis. To date, there is limited evidence regarding whether individuals with potential celiac disease face an increased risk of long-term complications compared to healthy individuals.

While celiac disease fulfills many of the WHO criteria for screening, uncertainties regarding cost-effectiveness, long-term benefits, and the management of potential celiac disease present significant challenges. Addressing these gaps will require efforts from researchers, policymakers, and healthcare providers to ensure that screening programs are both effective and sustainable. Until then, the ten criteria for mass-screening for celiac disease cannot be considered met.

In the future, the WHO may play and important role by enhancing global awareness of celiac disease screening in children by developing evidence-based guidelines, advocating for policy integration, and supporting healthcare provider training. Additionally, WHO can promote research, facilitate data sharing, and incorporate screening into existing child health programs to improve early diagnosis and patient outcomes worldwide.

## 4 Summary

Celiac disease meets many of the WHO criteria for public health screening, including high prevalence, availability of a suitable diagnostic test, and the existence of an effective treatment. Early detection through screening could potentially improve health outcomes by preventing complications like growth retardation, osteoporosis, and gastrointestinal malignancies. However, the current evidence is not conclusive regarding the overall benefit of mass screening. More research is needed to evaluate the long-term benefits of early detection, especially in asymptomatic individuals and those with potential celiac disease, optimize screening strategies, and assess the cost-effectiveness of screening programs. Until further evidence becomes available, clinicians should have a low threshold for testing for celiac disease in individuals with gastrointestinal symptoms, extraintestinal manifestations or laboratory finding associated with celiac disease, and screening strategies should focus on high-risk groups, such as first-degree relatives of patients with celiac disease and those with related autoimmune conditions.
